# Multiple thoracic intramedullary schwannoma: A case report

**DOI:** 10.1016/j.ijscr.2024.109330

**Published:** 2024-02-01

**Authors:** Novan Krisno Adji, Komang Yunita Wiryaning Putri, Laksmi Indreswari, Rudy Gunawan, Muhammad Yuda Nugraha

**Affiliations:** a Department of Neurosurgery, Soebandi Regional Hospital, Jember, Indonesia; b Department of Neurology, Soebandi Regional Hospital, Jember, Indonesia; c Department of Surgery, Soebandi Regional Hospital, Jember, Indonesia; d Faculty of Medicine, University of Jember, Indonesia

**Keywords:** Case report, Gross total resection, Intramedullary, Schwannoma, Spine surgery

## Abstract

**Introduction and importance:**

Schwannoma's are benign but clinically progressive tumours. Mostly, they present as intradural extramedullary and as a single lesion. They are quite rare in the intramedullary region and multiple lesions. We report a rare case of Multiple Intramedullary Schwannoma in the thoracic region. The aim of this study to inform an uncommon case of intramedullary schwannoma and support an appropriate preoperative diagnostic.

**Clinical presentation:**

A 43-year-old female patient was admitted with gradual onset weakness of both lower limbs (4/2) for last two months. Magnetic resonance imaging (MRI) scan disclosed an intramedullary tumour at the thoracal 11th and 12th vertebral levels. It measured 30x20x15 mm and 20x20x12 mm. Complete total resection of multiple lesions was done. Schwanoma's was confirmed based on the histopathological finding. The patient was discharged on 4th day post operative with both leg power 5/5 and needed to medical rehabilitation. Follow-up examination 1 months after surgery revealed favourable, neurological condition (modified McCormick scale: grade I).

**Clinical discussion:**

Intramedullary schwannoma is often misdiagnosed as other types of intramedullary tumour. Schwannomas are usually benign and have well defined cleavage plane. Total resection achievable in most cases, offers the best clinical outcome and avoids subsequent recurrence.

**Conclusion:**

Preoperative diagnosis of intramedullary schwannoma will help establish the optimum medical and surgical treatment and the prognosis. Timely surgery before permanent neurological deficit and gross total resection is recommended to achieve good clinical outcome.

## Highlights

• Schwannoma’s are benign but clinically progressive tumors and mostly present as intradural extramedullary and as a single lesion
• We report a rare case of Multiple Intramedullary Schwannoma in the thoracic region.
• Preoperative diagnosis of intramedullary schwannoma will help establish the optimum medical and surgical treatment and the prognosis.

## 1 Introduction

Schwannomas account for 30 % of the primary spinal cord tumours [1,2]. They are usually intradural-extramedullary (IDEM) and/or extradural in location, and the diagnosis of this tumour is usually not difficult when based on magnetic resonance imaging (MRI) results [3]. These tumours are slow growing benign spinal nerve sheath tumours which are diagnosed by imaging studies or by progressive neurological deficit. These tumours mostly present in 4th to 6th decades in life [4]. However, schwannomas rarely occur in an intramedullary location, and intramedullary schwannomas represent only 0.3 %–1.5 % of the primary intraspinal tumours [1]. A solitary intramedullary schwannoma is the most common presentation [5] There were six cases of multiple intramedullary schwannoma, but there was still no report about incidence of this rare condition in thoracic spinal cord [3]. We report a rare case multiple intramedullary Schwannoma of the thoracic spinal cord. This case report was written by following the Surgical Case Report (SCARE) guidelines [6,7].

## 2 Case presentation

A 43-year-old female patient was admitted with gradual onset weakness of both lower limbs for last two months. She had no traumatic accident, no other previous disorders such as neurofibromatosis or specific skin lesions, and with no comorbid.

### 2.1 Clinical presentation

Neurological examination revealed hypoesthesia and paresis both lower limbs. All modalities of sensation were absent below L1 dermatomal level. There was decreasing in the motor power of both legs. The left leg was 4 while the right leg was 2 with flexor spasm. The bilateral knee jerks and ankle jerks were hyperactive and well sustained ankle clonus was observed. The patient was defined as grade 4 according to the modified McCormick Scale.

### 2.2 Neuroimaging finding

**Fig. 1:**
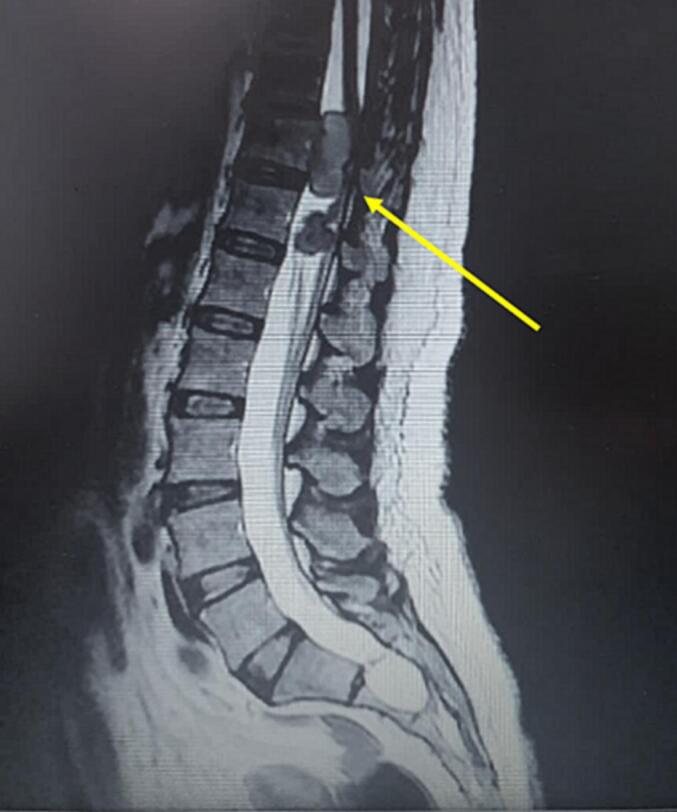
MRI Sagittal section showed multiple intramedullary tumours at the thoracal 11th and 12th vertebral levels. It measured 30 mm × 20 mm and 20mmx15mm.

Magnetic resonance imaging (MRI) scan disclosed an intramedullary tumour at the thoracal 11th and 12th vertebral levels. It measured 30 mm × 20 mm and 20mmx15mm (Fig. 1). This lesion was well defined. It was isointense on T1-weighted images and hyperintense on T2W images. Marked homogeneous contrast enhancement was noted.

### 2.3 Operative finding

**Fig. 2:**
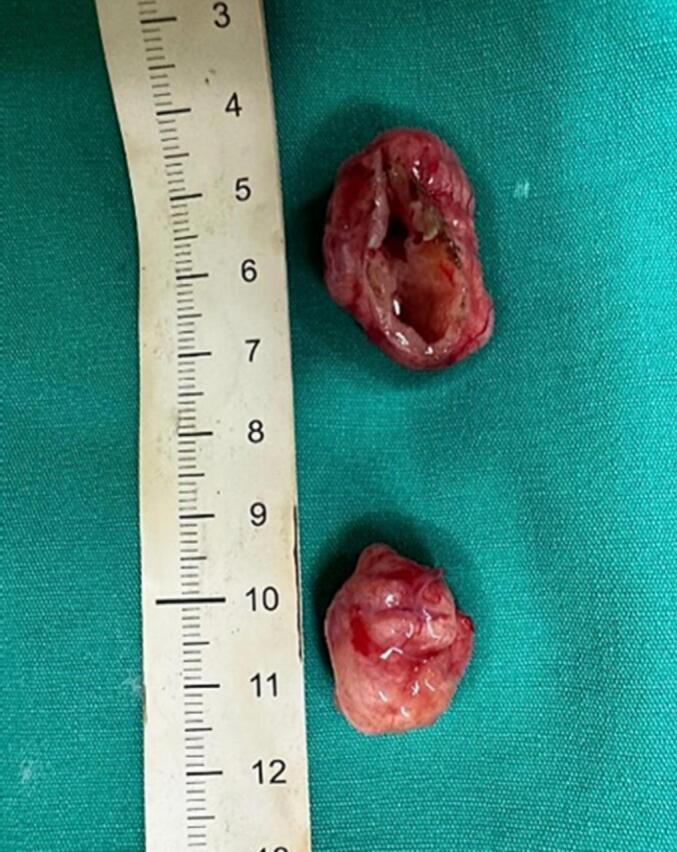
The tumours appeared as a solid, yellowish mass comparable with a schwannoma.

The operation was done with standard posterior middle approach. No oedema was seen in the spinal cord. Laminoplasty T11-T12 was performed and dura opened in the midline. After a median myelotomy, complete total resection of multiple tumours was done. Intraoperatively, there was two tumours (measuring 30x20x15 mm and 20x20x12 mm) appeared as a solid, yellowish mass and there was a campsule comparable with a schwannoma (Fig. 2). The nerve roots were not involved by the tumour.

### 2.4 Post-operative recovery

Immediately after the surgery, the sensory and motor functions of the patient were intact. During the hospital stay, the sensory and motor function developed gradually. The patient was discharged on 4th day post operative with both leg power 5/5 and needed to medical rehabilitation. Follow-up examination 1 months after surgery revealed favourable, neurological condition (modified McCormick scale: grade I) [8].

### 2.5 Histopathological finding

**Fig. 3:**
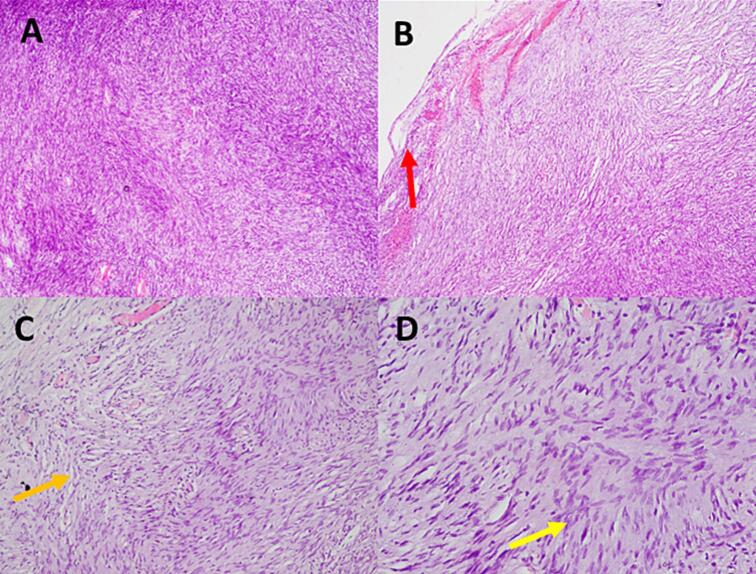
A. Antonie A and B area; B. Capsule of the tumour; C. Verocay bodies; D. Spindle wavy cell.

There are hypercellular areas and hypocellular areas (Antonie A and B) (Fig. 3A). There was a capsule in the outside of the tumour tissue (Fig. 3B). In the hypercellular area consists of spindle-shaped cells (wavy cells) that appear partially palisaded (verocay bodies) (Fig. 3C-D).

## 3 Discussion

Schwannoma, also known as a Schwann cell tumour, primarily affects the comparatively superficial regions of the head, neck, trunk, and limbs [9]. Schwannomas account for about 30 % of the primary spinal cord tumours. Most schwannomas are IDEM or extradural with a dumbbell extension. Intramedullary schwannomas are very rare, accounting for only 0.3 % of all intraspinal tumours and 1.1 % of all spinal cord schwannomas [1]. Majority of cases of intramedullary schwannoma have been reported in the cervical spine (63 %) followed by thoracic (26 %) and lumbar segments (11 %) of spinal cord [10,11]. Intramedullary schwannoma is known to predominate in males (male: female = 3:1) and the mean age of symptom onset is the fourth decade of life [2,10].

According to new WHO classification of tumours, there are three types of shwannomas; cellular, plexiform, and melanotic [12]. There is no correlation between the classification of Antoni and the MRI findings [10]. Most of the pathological findings were Antoni type A or some mixture with Antoni type B [13]. Histologically these tumours are composed of two types of growth patterns termed as Antoni A and Antoni *B. Antoni* A areas are hypercellular showing spindle shaped cells in the collagenous background. Distinctive nuclear palisading in which parallel rows of nuclei are arranged with intervening eosinophilic zone termed as verocay bodies. Antoni B areas are loose and often have mucinous or myxoid background with stellate cells. Immunohistochemically tumour cells are positive for S-100 protein [4] and non-reactive to glial fibrillary acidic protein (GFAP) and epithelial membrane antigen (EMA) [13]. Unlike gliomas or other malignant lesions, schwannomas are benign, posteriorly located and have a well-defined plane of cleavage which makes its complete excision achievable resulting in the best clinical outcome avoiding potential chances of recurrences. Also, its rare occurrence makes it difficult to differentiate from others preoperatively or during the surgery. Therefore, an intraoperative histologic examination would help to confirm the diagnosis and adopt subsequent complete excision thus influencing the overall management significantly [14].

The pathogenesis of IMS is controversially debated among experts because of the absence of Schwann cells within the central nervous system (CNS) in healthy individuals. The origin of the tumour has multiple factors. Currently, there are six hypotheses regarding the origin of IMS: (a) conversion of pial mesodermal cells into neuroectodermal Schwann cells; (b) migration and late neoplastic growth of ectopic Schwann cells during embryonal development; (c) origin from Schwann cells from the perivascular nerve plexus surrounding the blood vessels within the CNS; (d) schwannosis in proximity to the anterior spinal artery; (e) centripetal growth from a dorsal nerve root entry zone into the spinal cord and (f) result from imperfect regeneration of the spinal cord after mechanical trauma or chronic disease [3].

The symptoms of intramedullary spinal schwannoma were sensory and motor dysfunctions, which eventually appeared in the late stages of lesion progression. No tumours could be differentiated from common intraspinal tumours. However, somatic and root pain were the initial major complaints in patients with schwannoma [15].

Intramedullary schwannoma has no specific imaging features. However, the diagnosis of schwannoma should be considered if an intramedullary tumour has a clear boundary in the spinal cord and intense enhancement based on MRI [16]. MRI is the modality of choice for diagnosing intraspinal tumours. Intramedullary schwannomas presented with two patterns: solid lesions without a cystic portion; and cystic-solid lesions with associated cyst formation. The solid portion was isointense to hypointense on T1-weighted images. T2-weighted images usually showed hyperintense signal, with occasional isointense or low-signal areas. Segmental fusiform dilatation of the cord is common, and peritumoral edema, which is usually present in astrocytoma, is uncommon. Contrast-enhanced T1-weighted images better delineate the lesion and differentiate solid from cystic components and edema [2].

Owing to its low incidence and lack of clinical and imaging manifestation, intramedullary schwannoma is often misdiagnosed as other types of intramedullary tumour such as ependymoma, astrocytoma, hemangioblastoma and subependymoma, among others [17].

Unlike the more malignant lesions of cord like glioma, schwannomas are usually benign and have well defined cleavage plane. This makes total resection achievable in most cases, offers the best clinical outcome and avoids subsequent recurrence. Hence, the preferred mode of treatment for IM schwannoma is gross total surgical resection to an extent as much as possible, without causing any neurologic deficit [4,18]. However, in certain infiltrative variants of intramedullary schwannoma, this may not be possible. When the tumour is adherent to neural tissue, sub-total resection is likely to improve neurological function followed by a second surgery later or adjuvant radiotherapy. The role of adjuvant radio therapy is not well established [11].

Conventional Schwannomas mostly have a good prognosis, with less than 5 % recurrence reported in literature. However, data on long term follow up of intramedullary schwannoma is lacking and highlighting the need for long term follow up in this group of patients [11].

## 4 Conclusion

Intramedullary schwannomas are benign but clinically progressive lesions. The accurate diagnosis depends on pathology. In order to achieve good functional results, it is very important to perform timely surgery before permanent neurological deficit occurs. When gross total resection cannot be achieved, subtotal resection for decompression is advised. Postoperative radiotherapy is not recommended for these benign tumours. Clinic and radiological follow-up are required to evaluate if there is regrowth of the residual tumour.

## Patient consent

Written informed consent was obtained from the patient for publication and any accompanying images. A copy of the written consent is available for review by the Editor-in-Chief of this journal on request. Written informed consent was obtained from the patient and family.

## Ethical approval

Ethical approval for this study (Ethical Clearence Number 440/16083/610/2023) was provided by the xxxxxxxx on November 14, 2023. Written consent was obtained from the patient's family.

## Funding

No funding was applied for this study.

## Author contribution

NKA and KYWP contributed to the design of this study. RG, MYN contributed to data collection and analysis. NKA, LI, RG, MYN contributed to writing the article, and critical revision, and all approved the final manuscript.

## Guarantor

Novan Krisno Adji.

## Research registration number

N/A.

## Conflict of interest statement

The authors declare no conflict of interest.

## Data Availability

Not applicable.
